# Performance of GPT-3.5 and GPT-4 on standardized urology knowledge assessment items in the United States: a descriptive study

**DOI:** 10.3352/jeehp.2024.21.17

**Published:** 2024-07-08

**Authors:** Max Samuel Yudovich, Elizaveta Makarova, Christian Michael Hague, Jay Dilip Raman

**Affiliations:** 1 Department of Urology, Penn State Health Milton S. Hershey Medical Center, Hershey, PA, USA; 2 Penn State College of Medicine, Hershey, PA, USA; Hallym University, Korea

**Keywords:** Artificial intelligence, Certification, Graduate medical education, United States, Urology

## Abstract

**Purpose:**

This study aimed to evaluate the performance of Chat Generative Pre-Trained Transformer (ChatGPT) with respect to standardized urology multiple-choice items in the United States.

**Methods:**

In total, 700 multiple-choice urology board exam-style items were submitted to GPT-3.5 and GPT-4, and responses were recorded. Items were categorized based on topic and question complexity (recall, interpretation, and problem-solving). The accuracy of GPT-3.5 and GPT-4 was compared across item types in February 2024.

**Results:**

GPT-4 answered 44.4% of items correctly compared to 30.9% for GPT-3.5 (P<0.00001). GPT-4 (vs. GPT-3.5) had higher accuracy with urologic oncology (43.8% vs. 33.9%, P=0.03), sexual medicine (44.3% vs. 27.8%, P=0.046), and pediatric urology (47.1% vs. 27.1%, P=0.012) items. Endourology (38.0% vs. 25.7%, P=0.15), reconstruction and trauma (29.0% vs. 21.0%, P=0.41), and neurourology (49.0% vs. 33.3%, P=0.11) items did not show significant differences in performance across versions. GPT-4 also outperformed GPT-3.5 with respect to recall (45.9% vs. 27.4%, P<0.00001), interpretation (45.6% vs. 31.5%, P=0.0005), and problem-solving (41.8% vs. 34.5%, P=0.56) type items. This difference was not significant for the higher-complexity items.

**Conclusions:**

ChatGPT performs relatively poorly on standardized multiple-choice urology board exam-style items, with GPT-4 outperforming GPT-3.5. The accuracy was below the proposed minimum passing standards for the American Board of Urology’s Continuing Urologic Certification knowledge reinforcement activity (60%). As artificial intelligence progresses in complexity, ChatGPT may become more capable and accurate with respect to board examination items. For now, its responses should be scrutinized.

## Graphical abstract

[Fig f3-jeehp-21-17]

## Introduction

### Background and rationale

Chat Generative Pre-Trained Transformer (ChatGPT) is a widely used large language model that has gained worldwide popularity since its release in November 2022. ChatGPT consists of a chatbot interface that generates verbal responses to a wide variety of prompts by producing a sequence of words based on previously input text. There are 2 available versions at the time of this manuscript: GPT-3.5 and GPT-4. The former is freely available, while the latter is restricted to a subscription model with time-gated limitations on its use. GPT-4 is thought to be a significant improvement over GPT-3.5 with respect to the speed and accuracy of responses. At the time of our present study, both were trained with a substantial dataset of information available on the internet through September 2021. Practical applications of the model have been made in numerous fields, including medicine. Within urology, ChatGPT has been used to manage clinical scenarios and answer items [1].

The Self-Assessment Study Program (SASP) is an educational tool jointly created and delivered by the American Board of Urology (ABU) Examination committee and the American Urological Association (AUA). This educational product is widely used by urology residents to prepare for oral board examinations and the annual urology in-service examination. Additionally, the SASP serves in part as an item bank for the ABU Continuing Urologic Certification (CUC) knowledge reinforcement activity [2].

Annually, item sets consisting of 150 multiple-choice items are released. Previous studies involving ChatGPT investigated its accuracy with the 2021 and 2022 SASP item sets. One study showed that GPT-3 (an earlier version of ChatGPT than those used in the present study) answered 42% correctly for SASP 2021 and 30% correctly for SASP 2022, suggesting a potential trend of increased accuracy of the language models for earlier iterations of SASP [1].

### Objectives

The aim of our study was to characterize the accuracy of GPT-3.5 and GPT-4 over a larger sample size of standardized urology items and to determine whether there is a significant benefit to the use of GPT-4 over GPT-3.5 for answering these items. Such observations may have implications for the proposed CUC program knowledge reinforcement activity, which is administered as a self-paced, open resource examination.

## Methods

### Ethics statement

No human subjects or healthcare data were used in this investigation, and ethical approval was not needed.

### Study design

This descriptive study investigated the performance of GPT-3.5 and GPT-4 on urology board exam-style items.

### Setting

We created 700 unique multiple-choice board exam items that were in the style of—but wholly distinct from—standardized SASP program items. This approach was specifically used to avoid any degree of copyright infringement of content produced either by the AUA or the ABU. The item content was reviewed and sorted into the following categories: urologic oncology, endourology or kidney stone disease, sexual medicine or infertility, pediatric urology, reconstruction or trauma, and neurourology. Each item was standardized to resemble the format of an item stem followed by 5 letter-labeled answer choices. Representative questions from each content category and level of complexity are provided in Supplement 1.

### Variables

The primary outcome measured was the accuracy of GPT-3.5 and GPT-4 with respect to answering multiple-choice items.

### Data sources and measurement

**Fig. 1.**
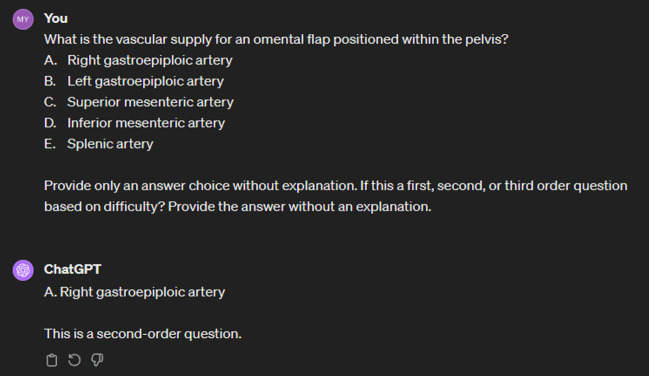
Example of a multiple-choice item and prompt submitted to ChatGPT, along with the response.

The accuracy of GPT-3.5 and GPT-4 was evaluated for every multiple-choice item in February 2024. For each item, a new session of GPT-3.5 was opened. One item per session was copied and pasted into the chat box followed by the prompt: “Provide only an answer choice without explanation” (Fig. 1). The answer choice provided by ChatGPT was then compared to the correct answer determined by the study authors, and a binary result was recorded to reflect whether the answer choice did or did not coincide with the correct answer. No items required additional clarification to retrieve an answer. The same process was repeated with GPT-4. Item complexity was determined using additional prompting after the answer choices were provided by ChatGPT. This was requested using the prompt: “Is this a first, second, or third order item. Provide the answer without an explanation.” First-order items were defined to be recall of factual information. Second-order items involved interpretation of data for diagnostic purposes. Third-order items required the interpretation and integration of information for problem-solving, management, or prognostic purposes.

### Bias

Each multiple-choice item was submitted to ChatGPT using a new chat session in order to avoid answer contamination across items.

### Study size

Two generative artificial intelligence platforms, GPT-3.5 and GPT-4 were used to assess a total of 700 urology items.

### Statistical methods

Statistical analysis was performed using the R software suite, and the chi-square test was used to compare the performance of GPT-3.5 and GPT-4 according to item complexity and item category. A 5% significance level was used.

## Results

A total of 700 unique items were independently created by the study authors in the style and taxonomy of SASP items with respect to accuracy and complexity. The distribution of items according to topic domains was as follows: 192, urologic oncology; 71, endourology or kidney stone disease; 79, sexual medicine or infertility; 85, pediatric urology; 62, reconstruction or trauma; and 51, neurourology items. A total of 340 recall, 305 interpretation, and 55 problem-solving items were evaluated (Dataset 1).

**Fig. 2.**
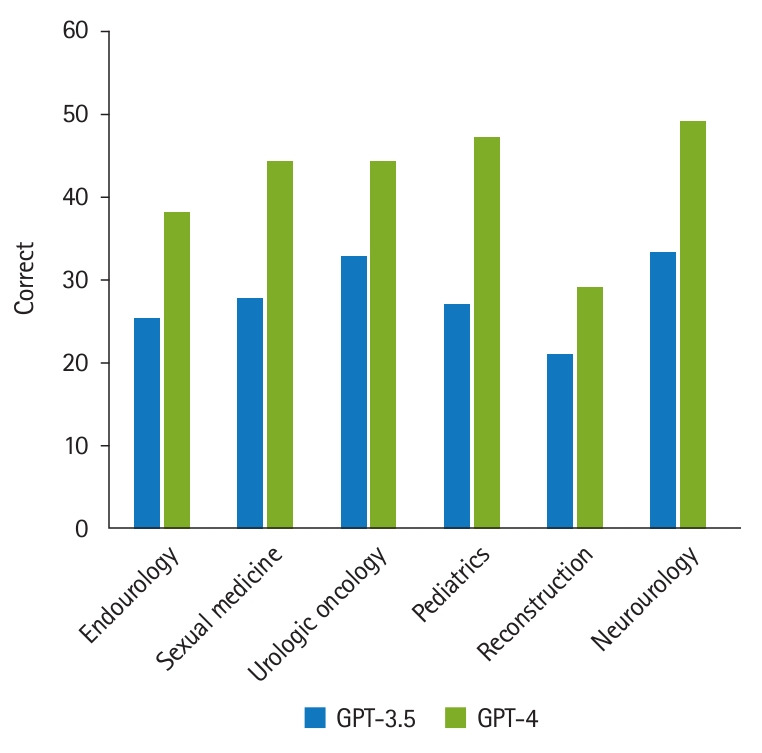
Percentage of items correct by GPT-3.5 and GPT-4 by item category. ^*^P<0.05 (statistically significant).

GPT-3.5 answered 29.7% and GPT-4 answered 45.5% of items correctly (P<0.00001). GPT-4 performed more accurately than GPT-3.5 on items across all 3 complexity levels, rising to the level of statistical significance for recall (P<0.0001) and interpretation (P=0.005) items (Table 1). GPT-4 answered more items correctly across all six content categories; however, this was only statistically significant in the categories of sexual medicine (P=0.046), urologic oncology (P=0.028), and pediatric urology (P=0.012). These findings are graphically represented in Fig. 2.

We found that GPT-3.5 and GPT-4 both performed inconsistently with respect to each other. The proportion of items correctly answered by GPT-3.5 and incorrectly by GPT-4 was 9.1%. Twenty-five percent of items were answered correctly by only GPT-4. Forty-five percent of items were answered incorrectly by both versions, and 20.6% were answered correctly by both versions.

## Discussion

### Key results

GPT-4 outperformed GPT-3.5 when answering multiple-choice urology items; however, the accuracy was below passing rates for urology certification testing.

### Interpretation

In this study, we show that GPT-4 and GPT-3.5 are capable of answering board exam-style urology items, although their accuracy remains poor. Deebel and Terlecki investigated the accuracy of GPT-3 with respect to SASP items from 2021 and 2022 [1]. Their study found the percentage correct to be 42.3% and 30.0% for the 2021 and 2022 respectively. The accuracy for 2022 was similar to our overall accuracy of 29.7% for GPT-3.5; however, this was achieved using a slightly more updated version of ChatGPT.

Later generations of ChatGPT have been touted as improving with respect to item accuracy for other standardized exams, such as the MCAT and USMLE [3,4]. GPT-4 has specifically been studied for significant improvements in accuracy. Out study found that GPT-4 answered significantly more items correctly than GPT-3.5. GPT-4 also did not consistently correctly answer items that were correctly answered by ChatGPT 3.5, as approximately one-tenth of all items were answered correctly by ChatGPT 3.5 and incorrectly by GPT-3.5. Overall, the higher-performing GPT-4 would still not achieve the proposed minimum passing standards for the ABU CUC knowledge reinforcement activity of 60% [2]. Prior work involving ChatGPT and urology assessment items detailed inconsistencies in answers or failures to produce a specific answer choice. Cocci et al. [5] discussed the importance of wording prompts to produce a definitive answer. In our study of 700 items, there were no instances in which ChatGPT yielded ambivalent answers. We attribute this finding to the direct wording of the prompt, which forces ChatGPT to provide a single answer choice.

### Comparison with previous studies

There has been significant variability among surgical subspecialties with respect to standardized written board exam items. Ali et al. demonstrated passing rates for mock neurosurgical board exam items and accuracy that were similar between GPT-4 and item bank users [6]. ChatGPT was able to exceed trainee performance on thoracic surgery items and achieve passing rates [7]. In orthopedic surgery, plastic surgery, and ophthalmology, ChatGPT performed poorly on practice board exam items [8-10]. One study investigating the South Korean general surgery board exams found a significant improvement in accuracy in GPT-4 compared to GPT-3.5, which was not consistently replicated by our study [11].

### Limitations

One limitation of this study involves changes to ChatGPT’s accuracy over time. Chen et al. [12] demonstrated that GPT-3.5 and GPT-4 have variable accuracy over time. When asked the same items in March 2023 and June 2023, GPT-4 was less accurate at identifying prime numbers in June 2023 than in March 2023; however, the contrary was true for GPT-3.5. The quality of responses also worsened in both models for code generation, and GPT-4 was less willing to answer sensitive items in June 2023 than in March 2023 [12]. Our data were collected in February 2024 and may reflect a decline in the accuracy of GPT-4. Language models such as ChatGPT are believed to be trainable from the prompts provided to them. It is therefore possible that recent studies involving the 2021 and 2022 SASP item banks may have inflated the accuracy of the responses.

### Generalizability

A significant implication of the ability of artificial intelligence to accurately answer multiple-choice items is related to the recertification process of board-certified practicing urologists. The ABU is spearheading a transition of recertification to a program known as the CUC [2]. This will involve formative assessments in which at least 30% of items will be derived from the last 5 years of the SASP in an open-resource fashion. Additional items will be derived from clinically beneficial journal articles. The benefit of these assessments is that candidates can research topics and learn from the literature in a form of knowledge reinforcement. Candidates enrolled in CUC will be expected to achieve accuracy thresholds of at least 60% or 80% depending on the stage of certification, neither of which is presently achievable using ChatGPT. It is possible that as ChatGPT improves, these items may be answerable by artificial intelligence without significant knowledge gain on the part of the user, bypassing the learning experience.

### Suggestions

Our methodology excluded items that involved the interpretation of images, urodynamics, charts, or other visual data. These items tend to have higher complexity. Therefore, the omission of these items reduces the total number of available interpretation and problem-solving items available in the study. With the recent release of image interpretation in GPT-4, further study of these items may be possible.

### Conclusion

Our study demonstrates that although GPT-3.5 and GPT-4 are capable of answering multiple-choice standardized urology board exam items, their accuracy is inconsistent both overall and across categories. GPT-4 generally performs better than GPT-3.5; however, the accuracy of GPT-4 is poor compared to the minimum passing standard of the ABU CUC. These results show that although ChatGPT can confidently produce answers, those answers should be scrutinized by the reader for accuracy and cannot be wholly relied upon for the purposes of education or board certification.

## Figures and Tables

**Figure f3-jeehp-21-17:**
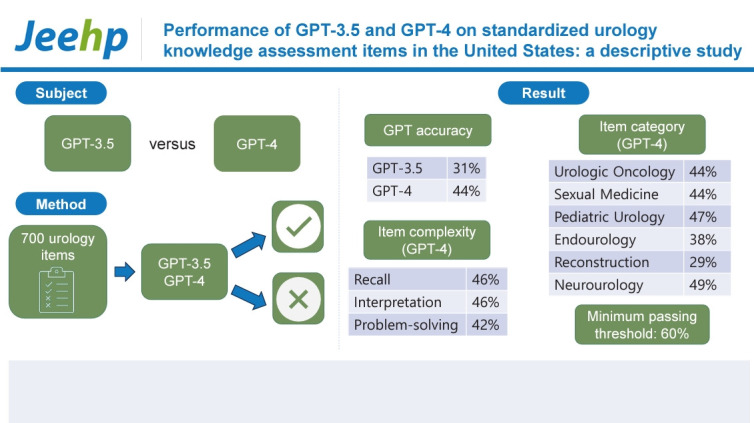


**Table 1.** Performance of ChatGPT on multiple-choice urology board exam-style items

	GPT-3.5	GPT-4	P-value
Total (n=700)	208 (29.7)	318 (45.4)	<0.00001
Category			
Urologic oncology (n=192)	64 (33.9)	84 (43.8)	0.028^*^
Endourology (n=71)	18 (25.7)	27 (38.0)	0.15
Sexual medicine (n=79)	22 (27.8)	35 (44.3)	0.046
Pediatrics (n=85)	23 (27.1)	40 (47.1)	0.012
Reconstruction (n=62)	13 (21.0)	18 (29.0)	0.41
Neurourology (n=51)	17 (33.3)	25 (49.0)	0.11
Complexity			
Recall (n=340)	93 (27.4)	156 (45.9)	<0.00001
Interpretation (n=305)	96 (31.5)	139 (45.6)	0.0005
Problem-solving (n=55)	19 (34.5)	23 (41.8)	0.56

Values are presented as number (%).

* P<0.05 (statistically significant).

## Supplementary materials

Supplementary files are available from Harvard Dataverse: https://doi.org/10.7910/DVN/4EJOCL

Supplement 1. Representative test items (correct answers bolded).

Supplement 2. Audio recording of the abstract.
