# Supplementary material for: Performance of GPT-3.5 and GPT-4 on standardized urology knowledge assessment items in the United States: a descriptive study
Source: J Educ Eval Health Prof. 2024 Jul 8;21:17. doi: 10.3352/jeehp.2024.21.17 (PMC11893186; doi:10.3352/jeehp.2024.21.17)
Supplement: Supplementary file 2 — Supplement 1. Representative test items (correct answers bolded). [file jeehp-21-17-suppl1.docx]

**Supplement 1.** Representative test items (correct answer bolded)

Recall type:

What is the vascular supply for an omental flap positioned within the pelvis?

**A. Right gastroepiploic artery**

B. Left gastroepiploic artery

C. Superior mesenteric artery

D. Inferior mesenteric artery

E. Splenic artery

Interpretation type:

A 7-month-old male has proximal hypospadias and left nonpalpable testis. The right testis is in the scrotum and palpable normal. Karyotype is 46,XY. Diagnostic laparoscopy is performed and shows an open internal ring with both vas deferens and gonadal vessels entering the ring. What is the most likely diagnosis?

A. Testicular agenesis

B. Testicular nubbin

**C. Undescended testis**

D. Streak gonad

E. Ovotestis

Problem-solving type:

A 65-year-old female with history of type II diabetes has bothersome urinary frequency, slow urinary stream, and recurrent urinary tract infections. Her exam shows no pelvic organ prolapse, decreased perineal sensation, and intact sphincter tone. Urinalysis is normal and post-void residual (PVR) is 350 mL. She is not willing to perform clean intermittent catheterization. The next step is:

A. Bethanechol

**B. Sacral neuromodulation**

C. Crede or Valsalva voiding

D. OnabotulinumtoxinA injection into urethral sphincter

E. Indwelling urethral catheter

Endourology:

What is the best management of 3 cm of asymptomatic seinstrasse?

**A. Extracorporeal shock wave lithotripsy**

B. Ureteroscopy

C. Percutaneous nephrolithotomy

D. Ureteral stent

E. Medical expulsive therapy

Sexual medicine:

A 45-year-old man with prior penile fracture has curvature and pain with intercourse. He has a palpable plaque with 75° of dorsal curvature. Which is the next step in management?

A. Stem cell therapy

**B. Collagenase injection**

C. Verapamil injection

D. Extracorporeal shock wave therapy

E. Observation

Urologic oncology:

A female patient is found to have a 2 cm cystic renal lesion with 6 Hounsfield units of attenuation. There are smooth, thin walls with non-enhancing septa and fine calcifications. What is the next step in management?

**A. No further imaging**

B. Ultrasound in 6 months

C. Computed tomography abdomen and pelvis in 1 year

D. Percutaneous biopsy

E. Partial nephrectomy

Pediatrics:

A 6-year-old boy with a history of posterior urethral valve resection as a neonate has persistent urinary incontinence and total urine volume of 3 L per day. The next step is:

A. Desmopressin

B. Imipramine

C. OnabotulinumtoxinA

**D. Timed, double voiding**

E. Reduce fluid intake

Reconstruction and trauma:

A patient sustains an electrical burn to the penis. Five hours later, the penile shaft and glans are erythematous with superficial sloughing and blistering. What is the next step in management?

A. Observation

B. Retrograde urethrogram

C. Urethral catheter

**D. Suprapubic tube**

E. Debridement

Neurourology:

A 78-year-old male has incontinence after transurethral resection of the prostate and radiotherapy for prostate cancer. PVR is low. Urodynamics demonstrate detrusor overactivity. What is the next step in management?

**A. Anticholinergics**

B. Alpha blocker

C. Male sling

D. Onabotulinumtoxin injection

E. Artificial urinary sphincter
